# Do High Frequency Ultrasound Images Support Clinical Skin Assessment?

**DOI:** 10.1155/2013/314248

**Published:** 2013-02-21

**Authors:** Alison P. Porter-Armstrong, Catherine Adams, Anne S. Moorhead, Jeannie Donnelly, Jane Nixon, Daniel L. Bader, Courtney Lyder, May D. Stinson

**Affiliations:** ^1^The Institute of Nursing and Health Research, School of Health Sciences, University of Ulster, Newtownabbey BT37 0QB, UK; ^2^School of Health Sciences, Faculty of Life and Health Sciences, University of Ulster, Newtownabbey BT37 0QB, UK; ^3^School of Sociology, Social Policy & Social Work, Queen's University Belfast, Belfast BT7 1LP, UK; ^4^Institute for Research in Social Sciences, School of Communication, University of Ulster, Newtownabbey BT37 0QB, UK; ^5^Tissue Viability, The Royal Hospital, Belfast Health and Social Care Trust, Belfast BT12 6BA, UK; ^6^Clinical Trials Research Unit, University of Leeds, Leeds LS2 9JT, UK; ^7^School of Engineering and Materials Science, Queen Mary University of London, London E1 4NS, UK; ^8^Faculty of Health Sciences, University of Southampton, Southampton SO17 1BJ, UK; ^9^School of Nursing, University of California Los Angeles, Los Angeles, CA 90095, USA

## Abstract

High frequency ultrasound imaging has been reported as a potential method of identifying the suspected tissue damage in patients “at risk” of pressure ulceration. The aim of this study was to explore whether ultrasound images supported the clinical skin assessment in an inpatient population through identification of subcutaneous tissue damage. Skin on the heels and/or sacral coccygeal area of fifty vascular surgery inpatients was assessed clinically by tissue viability nurses and with ultrasound pre operatively and at least every other day until discharge. Images were compared to routine clinical skin assessment outcomes. Qualitative classification of ultrasound images did not match outcomes yielded through the clinical skin assessment. Images corresponding to 16 participants were classified as subgroup 3 damage at the heels (equivalent to grade 2 pressure ulceration); clinical skin assessment rated no heels as greater than grade 1a (blanching erythema). Conversely, all images captured of the sacral coccygeal area were classified as normal; the clinical skin assessment rated two participants as grade 1b (non-blanching erythema). Ultrasound imaging is a potentially useful adjunct to the clinical skin assessment in providing information about the underlying tissue. However, further longitudinal clinical assessment is required to characterise images against actual and “staged” pressure ulceration.

## 1. Introduction 

A pressure ulcer is defined as an area of localised damage to the skin and the underlying tissue caused by prolonged mechanical loading involving a combination of pressure, shear, and/or friction [1] with costs to the individual including pain, embarrassment, social exclusion, and a reduced quality of life [2]. Financial costs to the NHS of this largely preventable condition have been estimated to range from *£*1.4 to *£*2.1 billion per annum [3]. One subset of pressure ulcers, known as deep tissue injuries, has been characterised by damage which is localised in tissues at the bone muscle fascia, and which progresses up through the tissues in the form of oedema until reaching the skin surface [4, 5]. These are not readily apparent to the eye, and thus, by the time the clinical signs of deep tissue injury are evident, the injury is often well established and its resulting prognosis is variable [6].

The need for the investigation into early detection of pressure ulcers so that timely healthcare interventions can occur has been recognised [7]. Early detection and prevention would greatly reduce the burden on the patient and the associated economic and social costs associated with this condition. A number of techniques capable of early detection have been proposed, including the ultrasonic visualisation of subcutaneous oedema [4, 7, 8]. High frequency ultrasound (HFUS), at a frequency of 20 MHz, allows the real-time two-dimensional imaging of internal structures in a noninvasive manner and has been shown to be a potentially valuable method for the assessment of subcutaneous tissue damage pertaining to numerous pathophysiologies including the evaluation of wounds [7, 9–11]. The potential it offers in establishing parameters associated with the echogenicity of the ultrasound image could provide earlier identification of tissue damage than that being achievable through clinical skin assessment or photography alone [7, 8].

The aim of this study was to explore whether ultrasound images supported clinical skin assessment in a cohort of vascular surgery hospital inpatients. 

## 2. Materials and Methods

Full ethical approval for the study was gained through the Office of Research Ethics Committee Northern Ireland (ORECNI). Hospital inpatients were approached to participate in the study if they were admitted for elective vascular surgery, provided written informed consent, and had intact skin on one or more areas to be scanned (sacral coccygeal area and either/or both heels). A single exclusion criterion of existing pressure damage of greater than, or equal to,* grade two *pressure ulceration (partial thickness skin loss involving epidermis, dermis, or both) [1] visible on the skin (including blisters, abrasions, and ulcers, where skin loss is present) of either heels or sacral coccygeal area was applied.

Patients were provided with written information on the study at a preassessment clinic approximately one week prior to admission and written informed consent gained within 24 hours of admission. During the six-month data collection period, a total of 90 patients were preassessed for vascular surgery, of whom 60 agreed to provide consent and were recruited to the study. Two participants subsequently withdrew consent, and a further eight participants were excluded from the analysis due to incomplete data resulting in a total of 50 participants being reported upon. Consenting participants had clinical assessments and high frequency ultrasound scanning conducted at least every other day throughout their inpatient hospital stay.

### 2.1. Clinical Assessments

Baseline clinical assessment was conducted by one of three tissue viability clinical research nurses (CRNs) using a comprehensive clinical research record Form (CRRF). Baseline characteristics were recorded including a summary of medical history and a number of completed standardised assessments including the Braden Scale for pressure ulcer risk [13], the Charlson index for comorbidities [14], and the Malnutrition Universal Screening Tool (MUST) for nutrition [15]. A clinical skin assessment was conducted at baseline, postoperatively and at least every other day by the CRN until discharge using a standard skin assessment record incorporating the modified EPUAP classification scale of pressure ulceration (Table 1) [12]. 

### 2.2. Instrumentation and Scanning Procedure

The EPISCAN I-200 high frequency ultrasound scanner (Longport Inc, USA) was used to capture the images in this study. All ultrasound assessments were conducted by two trained researchers. In order to minimize any participant discomfort caused by repeated moving and handling, images were recorded at the same time as clinical skin assessments being recorded by the CRN. Heels were scanned at the three areas of the greatest potential pressure, that is, lateral, posterior, and medial aspects as well as the bony prominences of the coccyx and right and left sacrums. 

### 2.3. Qualitative Image Assessment

**Figure 1:**
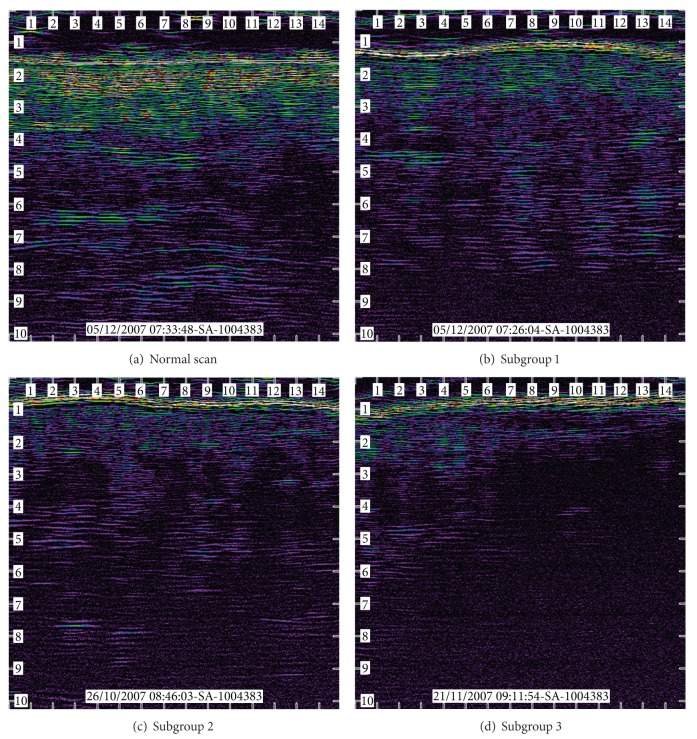
Examples of (a) “normal,” (b) “subgroup 1,” (c) “subgroup 2,” and (d) “subgroup 3” images.

Qualitative image assessment was performed by two blinded raters. Images were classified into four distinct subgroups based upon categorisation used by Quintavalle et al. [4] (Table 2 and Figure 1). 

Statistical analysis was performed using SPSS version 15. Data were found not to be normally distributed using the Kolmogorov-Smirnov test. An *α* level of *P* < 0.05 was set a priori for all the analyses. Friedman's tests were used to determine differences over time in the clinical skin assessments; Spearman's rank order correlations were applied to determine the relationship between the clinical skin assessment and the qualitative image analysis; and a weighted Kappa statistic was applied to the qualitative image analysis, results to determine the interobserver agreement. 

## 3. Results

Eleven females and 39 males with a mean age of 65 years (SD 9.66 years) participated in the study with average weight of 82.57 kgs (SD 17.41 kg), height of 171.74 cms (SD 9.13 cm), and body mass index of 28.14 (SD 4.38) Kg/m^2^. Whilst only three participants were considered to be “at risk” of pressure ulceration (Braden Scale score of 15–18) and 90% of participants were identified to be at low risk of malnutrition according to the “MUST” tool, more than half of the participants (56%) were within the high risk category of comorbidity as indicated by the Charlson index.

Of the 50 participants who completed the study, 32 had their heels and sacral coccygeal area scanned, 17 had heels only scanned, and one had only the sacral coccygeal area scanned. A total of 1492 ultrasound images were assessed by the two raters (Table 3). 

### 3.1. Clinical Assessment

No participants were clinically assessed as presenting with tissue changes greater than a grade 1b (nonblanching erythema of intact skin). Two participants were clinically assessed as showing signs of non-blanching erythema (*grade 1b*) on the marked skin sites on the coccyx, one of whom was also clinically assessed as showing signs of a *grade 1b* pressure ulcer on the left sacrum at a single time point postoperatively (Table 4). The two participants with nonblanching erythema had peripheral vascular disease and hypertension; however, neither of them were assessed as being “at risk” of developing pressure ulceration as per the Braden Scale [13].

Participants, heels were consistently clinically assessed as being either “normal” or presenting as no greater than a *grade 1a* (blanchable erythema of intact skin: see Table 4). Friedman's tests revealed no statistically significant changes over time.

### 3.2. Qualitative Image Assessment

Whilst clinical skin assessment identified the presence of non-blanching erythema on the coccyx in two participants and on the sacrum of one participant, all ultrasound images of the coccyx and sacrum for all participants were assessed as being “normal” by both raters. 

Conversely, 43 participants were assessed as having at least one heel image assessed by both raters as being *subgroup 1*, 34 having at least one heel image jointly assessed as being *subgroup 2*, and 16 having at least one heel image jointly assessed as being* subgroup 3 *(Table 3). The Friedman test conducted on the entire data set, and on the subsample of participants presented with subgroup 3 images, revealed no statistically significant changes over time. The weighted kappa statistic applied to the qualitative image classifications revealed an overall agreement of 0.80, indicating a good level of agreement between the raters for all images. 

## 4. Discussion

Increasing attention is being paid to the use of high frequency ultrasound scanning as a modality for wound and ulcer examination [9, 11] with some reports highlighting the potential application of high frequency ultrasound in imaging suspected subcutaneous oedema pertaining to pressure ulcers [7, 8, 10]. That is, in providing images of tissue damage below the skin surface that cannot be seen by the naked eye.

The present study investigated the use of high frequency ultrasound in supporting the clinical skin assessment by tissue viability nurses in the hospital inpatient setting. Two participants were clinically assessed as presenting with nonblanching erythema of intact skin (*grade 1b*) on the coccyx and for one individual on the sacrum. However, the images yielded by high frequency ultrasound for these participants were rated by both raters as being “normal.” That is, the internal structures in the images of the sacral coccygeal areas appeared clear, accompanied by good definition of the epidermis, dermis, and subcutaneous tissues. 

Conversely, the clinical skin assessment assessed no participant as presenting with skin damage greater than grade 1a (blanching erythema of intact skin) on any aspect of their heels, yet high frequency ultrasound revealed images concurrent with the suspected underlying tissue damage to the heels. The qualitative image analysis of this data set revealed 16 participants (32%) having at least one *subgroup 3* image. This incidence of *subgroup 3* category images, that is, sub-epidermal inflammation, strips of dermal damage, and major subcutaneous damage, infers the presence of clinical signs equivalent to *grade two* pressure ulceration and above. Such signs were not however assessed clinically. Due to the absence of any longer term followup following the discharge from the vascular surgery ward (average 5.8 days ± 3.38 days SD postadmission), it is unknown whether or not this suspected subcutaneous damage progressed into clinical signs of pressure ulceration. As the pathophysiology of the hypoechogenicity visualised in the *subgroup 3* images remains uncertain, and no correlation existed between the presence of those hypoechoic areas and the clinical assessment of pressure ulceration in this study, no firm conclusions can be drawn as to whether these images represented true deep tissue damage not visible to the eye through routine clinical skin assessment. With so little tissue damage clinically evident in this study, it remains unknown whether any areas of low echogenicity as visualised in the images equated to actual tissue damage.

## 5. Conclusion 

Ultrasound imaging offers a potentially useful adjunct to the clinical skin assessment in providing information about the underlying tissue damage not seen by the naked eye. However, further longitudinal clinical work is required to scan “at risk” individuals over time to characterise the images yielded against manifest pressure ulcers and the various stages of skin breakdown, as well as against clinical skin assessment outcomes. 

## Figures and Tables

**Table 1:** Modified European Pressure Ulcer Advisory Panel grading system [12].

Grade	Description
0	Intact skin with no visible erythema
1a	Blanchable erythema of intact skin
1b	Nonblanchable erythema of intact skin
2	Partial thickness skin loss involving epidermis, dermis, or both
3	Full thickness skin loss involving damage to or necrosis of subcutaneous tissue that may extend down to, but not through, the underlying fascia
4	Full thickness skin loss with extensive destruction, tissue necrosis, or damage to muscle, bone, or supporting structures

**Table 2:** Categories for the classification of high frequency ultrasound images [4].

Category	Description
0	Normal scan—no sign of PU development
Subgroup 1	Pockets of subcutaneous oedema
Subgroup 2	Strips of dermal damage and increased subcutaneous damage
Subgroup 3	Subepidermal inflammation, strips of dermal damage, and major subcutaneous damage
Ungraded	Unable to assess due to poor image quality

**Table 3:** Clinical Skin Assessment.

	Grade 0 (%)	Grade 1a (%)	Grade 1b (%)
Left lateral heel	45 (90.00)	19 (38.00)	0 (0)
Left posterior heel	45 (90.00)	18 (36.00)	0 (0)
Left medial heel	40 (80.00)	20 (40.00)	0 (0)
Right lateral heel^#^	44 (89.80)	20 (40.82)	0 (0)
Right posterior heel^#^	44 (89.80)	18 (36.73)	0 (0)
Right medial heel^#^	44 (89.80)	20 (40.82)	0 (0)
Coccyx*	30 (90.91)	5 (15.15)	2 (6.06)
Right sacrum*	31 (93.94)	4 (12.12)	0 (0)
Left sacrum*	32 (96.97)	5 (15.15)	1 (3.03)

^#^Data available for 49 participants; 1 participant had right above knee amputation.

*Total of 33 participants.

**Table 4:** High frequency ultrasound image assessment of total 1492 images.

	Number of images		Number of participants	
	Rater 1	Rater 2	Rater 1	Rater 2
0 (normal)	748	808	49	48
Subgroup 1	375	291	46	43
Subgroup 2	285	265	34	39
Subgroup 3	69	121	16	18
Ungraded	15	7	6	5
